# Effects of Juvenile Idiopathic Arthritis on Kinematics and Kinetics of the Lower Extremities Call for Consequences in Physical Activities Recommendations

**DOI:** 10.1155/2010/835984

**Published:** 2010-09-02

**Authors:** M. Hartmann, F. Kreuzpointner, R. Haefner, H. Michels, A. Schwirtz, J. P. Haas

**Affiliations:** ^1^Department of Motion Analysis, German Center for Pediatric and Adolescent Rheumatology, 82467 Garmisch-Partenkirchen, Germany; ^2^Department of Biomechanics in Sports, Faculty of Sports and Health, Technische Universität München, 80809 Munich, Germany

## Abstract

Juvenile idiopathic arthritis (JIA) patients (*n* = 36) with symmetrical polyarticular joint involvement of the lower extremities and healthy controls (*n* = 20) were compared concerning differences in kinematic, kinetic, and spatio-temporal parameters with 3D gait analysis. The aims of this study were to quantify the differences in gait between JIA patients and healthy controls and to provide data for more detailed sport activities recommendations. JIA-patients showed reduced walking speed and step length, strongly anterior tilted pelvis, reduced maximum hip extension, reduced knee extension during single support phase and reduced plantar flexion in push off. Additionally the roll-off procedure of the foot was slightly decelerated. The reduced push off motion in the ankle was confirmed by lower peaks in ankle moment and power. The gait of JIA-patients can be explained as a crouch-like gait with hyperflexion in hip and knee joints and less plantar flexion in the ankle. A preventive mobility workout would be recommendable to reduce these restrictions in the future. Advisable are sports with emphasis on extension in hip, knee, and ankle plantar flexion.

## 1. Introduction

Juvenile idiopathic arthritis (JIA) in children and adolescents is a chronic autoinflammatory affection which might occur in any joint [1]. The disease causes pain that may lead to posture and movement modifications and arouses muscular imbalance with reduced range of motion in the affected joints [2]. These processes may lead to malpositioning or compensatory movements that increase the risk for subsequent degenerative joint diseases. In previous studies an inflammation in joints of the lower extremity was associated with changes in the normal gait [3–5].

A method to analyze the human gait is the 3d-gait analysis. At the German Center for Pediatric and Adolescent Rheumatology Garmisch-Partenkirchen, this technique is performed in JIA patients with multiple affected joints in the lower extremities to quantify kinematics and kinetics to individualize and optimize the physio- and sports therapy. 

Physical activities are increasingly considered as an important part of treatment for JIA patients. Moreover inactivity is expected as a major factor of substantial negative effects for the musculoskeletal system, the whole body- composition, and the physical ability of JIA patients. The aim of therapy is to retain or regain an adequate level of activity in order to counteract the loss of coordination and fitness in spite of the disease activity.

In therapy of JIA, prevention of joint dysfunction and reeducation of physiological movements are important issues which might be supported by sport activities. 

The aim of this study was to compare the gait of JIA patients with normal gait and further to quantify malpositions in the lower extremities and joint restrictions during walking condition. These data may lead to recommendations about sport activities.

## 2. Methods

The study is based on a retrospective analysis of JIA patients admitted in the German Center for Pediatric and Adolescent Rheumatology, Garmisch-Partenkirchen between August 2006 and November 2009. The 3d gait analysis is a part of the routine procedures used to quantify movement restrictions to individualize physiotherapy. Written consent was delivered by the participants or parents (legal guardians) for the anonymous use of these data for scientific purpose.

### 2.1. Participants

The patient group (JIA-P) (*n* = 36) included children and adolescents, who suffered from JIA with symmetric polyarticular joint involvement with inflammation and/or movement restrictions in both hip, knee and ankle joints (sex: ♀ = 22; ♂ = 14; age: 13.2 ± 4.2 y; weight: 44.0 ± 17.7 kg; height: 1.49 m ± 0.15 m). For comparison a control group (CG) of 20 voluntary, healthy young individuals have been examined (sex: ♀ = 17, ♂ = 3; age: 17.9 ± 6.5 y; weight: 53.8 ± 15.0 kg; height: 1.59 ± 0.13 m).

Comparison of our measured standard values with results from the literature showed only minor deviations [6, 7]. Thus we have used our own CG to minimize the measurement error.

### 2.2. Data Collection and Processing

**Figure 1:**
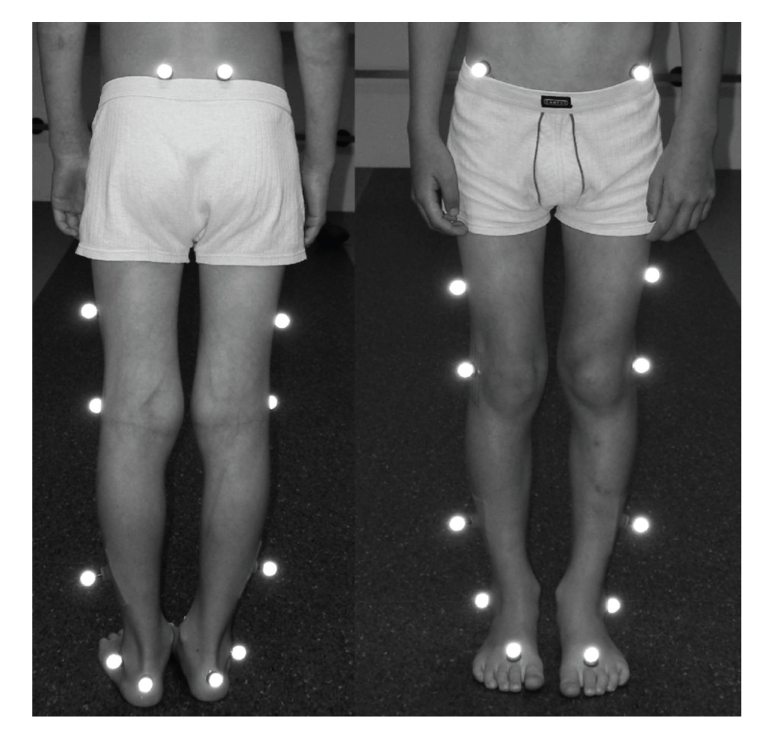
Placement of the Plug-in-Gait Model.

Gait analysis is performed in a 9 m long and 3 m wide laboratory which is equipped with a 3d-motion analysis system including six infrared cameras (120 Hz) (Vicon, MX3) and one 3d ground reaction force plate (1080 Hz) (AMTI). The participants were marked in accordance to the Plug-in-Gait Model for the lower extremities [8, 9] with 16 reflecting balls (Ø = 14 mm). This model supports calculation of joint angles, as well as moments and power with inverse dynamics (Figure 1).

As most of our JIA-patients had walking disabilities, we have asked the participants to select a walking speed which was pleasant for them. Each subject completed at least two attempts in order to get accustomed to the measuring situation before the analysis started. For the kinematic evaluation twelve left and right gait cycles were used. The kinetic data consist of three left and right steps of each individual. 

**Figure 2:**
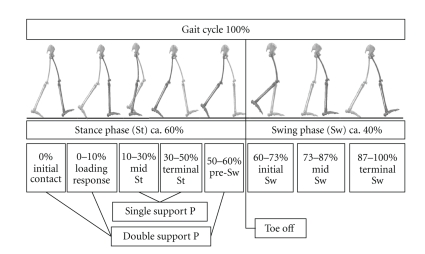
Normal gait cycle with approximated event timings (modified to Perry [6]).

The gait was scaled and normalized in separated gait cycles, consisting of a stance and a swing phase of one limb. A gait cycle starts with the initial contact and ends with the next initial contact of the same leg (Figure 2). A *t*-test for paired samples showed no significant differences within the investigation groups between the right and left side. Therefore the right and left results of each participant were averaged. In addition a clinical joint assessment was done by a physician according to the neutral zero method before 3d-gait analysis. Statistical analyses were made on the basis of arithmetic means (plus standard deviation) focused on the spatio-temporal parameters walking speed, step length, step width, and percentage time of foot off during one gait cycle. Due to the body height variation between the investigation groups, the step length (SL) and walking speed (*v*) were compared in their absolute value and additionally dimensionless after the scheme of Hof [10, 11] by using the leg length (*L*_leg_) and Newton's constant (*g*):

(1) $$\begin{array}{c} {\text{SL}}^{*}=\frac{\text{SL}}{L_{\text{leg}}},\\ v^{*}=\frac{v}{\sqrt{g*L_{\text{leg}}}}. \end{array}$$

The kinematic parameters of special interest were (i) pelvic tilt, (ii) pelvic obliquity, (iii) pelvic rotation, (iv) hip flexion/extension, (v) hip abduction/adduction, (vi) knee flexion/extension, (vii) ankle joint dorsal/plantar flexion, and (viii) plantar angle in order to describe the roll-off behavior of the foot (Figure 5). 

The maximum peak values of kinetic data were calculated in the ankle dorsal flexion moment and in the power that is generated in the ankle (Figure 6). While these data were normalized to the body weight, the ground reaction forces were presented in percent of body weight. The vertical force (*F* *z*) was compared in two peaks *F* *z*_1_ (loading response), *F* *z*_3_ (terminal stance phase) and in the valley *F* *z*_2_ (midstance phase) (Figure 7). The horizontal force in gait direction was analyzed in the maximum of the positive peak *F* *y*_1_ (deceleration effect) and in the minimum of the negative peak *F* *y*_2_ (acceleration effect) (Figure 7). 

Statistical analyses for comparison were performed using the *t*-test for two independent samples. All analyses have been performed bilaterally. The normal distribution was tested and proven by the kolmogorov-smirnov test for all parameters of interest [12]. The equality of variances was controlled by the Levene test. Statistical significance was determined at the level of *P* <  .05. SPSS 18.0 was used for statistics (SPSS Inc. USA).

## 3. Results

Kinematic and spatio-temporal data included all participants of JIA-P and CG. In five individuals (1 JIA-P, 4 CG) ground reaction force data were not available due to invalid contact or technical difficulties. Thus inverse dynamic calculations were possible only in 35 patients and 16 controls. Subsequently the account will focus predominantly on statistically significant differences, and conspicuous results of the joint assessment will be presented. 

### 3.1. Spatio-Temporal Parameters

Comparison of spatio-temporal parameters showed statistically significant differences in the self-chosen walking speed (*P*  <  .001). JIA-P went with an average speed of 1.06 m/s while CG had chosen 1.32 m/s. Nearly the same statistical significance was seen in the dimensionless comparison of the walking speed (*P*  <  .001). This decreased velocity in the patient group came along with a smaller step length absolutely (*P*  <  .001) as well as relative to leg length (*P*  <  .001). The step width was increased in JIA-P (0.12 m) compared to CG (0.09 m; *P*  <  .01). The foot off in JIA-P took place after 61.1% of gait cycle while in CG the foot off occurred at 59.9% (*P*  <  .05) (Table 1).

### 3.2. Kinematic Results

#### 3.2.1. Pelvis

**Figure 3:**
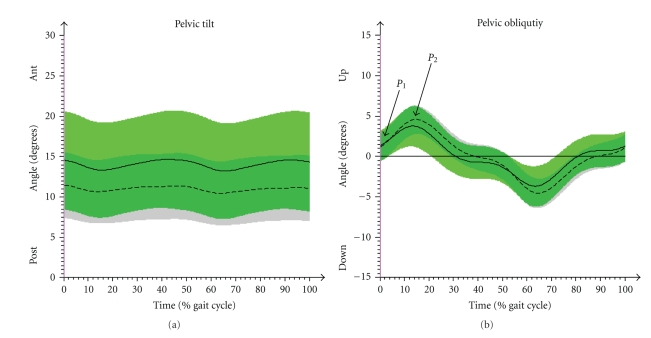
Comparison of angle progression in the pelvic tilt (a) and obliquity (b). CG (arithmetic mean & SD (- - -)); JIA-P (arithmetic mean and SD (—)). *P*_1_ (initial contact) and *P*_2_ (max. increase in loading response) are the points of interest in the pelvic obliquity.

Patients showed a stronger anterior tilting pelvis than controls (*P*  <  .05). The pelvic obliquity of JIA-P had a statistically significant smaller range of motion (ROM) in the contralateral drop of the pelvis from initial contact to maximum height during loading response (Figure 3) (*P*  <  .05). There were no statistically significant results in the ROM of pelvic rotation (Table 2).

#### 3.2.2. Hip

**Figure 4:**
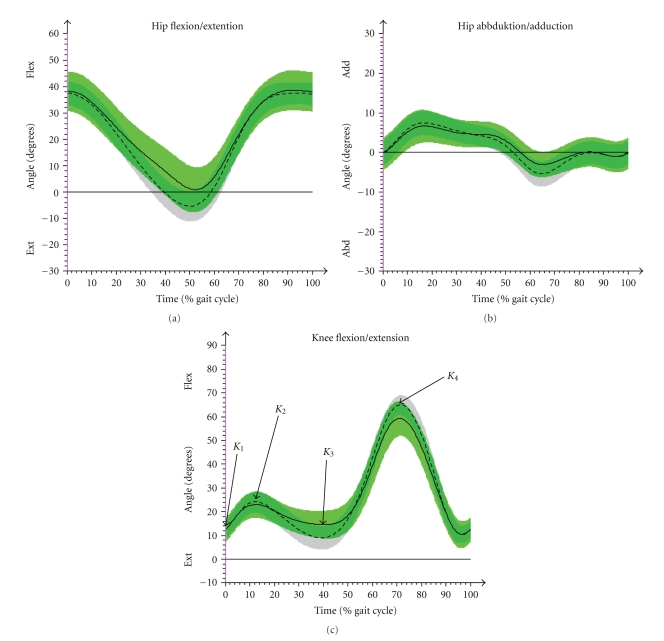
Results of gait analysis of CG (arithmetic mean & SD (- - -)) and JIA-P (arithmetic mean & SD (—)) in hip (a) flexion/extension, (b) abduction/adduction, and knee joint, (c) flexion/extension. *K*_1_ (initial contact), *K*_2_ (max. flex in loading response), *K*_3_ (max. extension in single support phase), and *K*_4_ (max. flexion in swing) are the points of interest in knee joint that were used for statistical analyses.

The maximum ROM during hip flexion and extension during stance phase appeared clearly different (*P*  <  .001) with JIA-P showing statistically significant lower values compared to CG (Table 2; Figure 4). While hip flexion was similar in both groups JIA-P performed minor hip extension (*P*  <  .01). The CG had a maximum extension average value of 5.8° while the JIA-P failed to reach full extension by 0.7° (flexion position). This corresponds to the results of the clinical assessment, where 12 of 36 patients showed bilateral hip flexion contractures. The maximum flexion position occurring either during landing phase or at the end of the swing-phase was increased in JIA-P, but however this was not significant. Abduction and adduction were measured as the ROM from neutral position to peak adduction or peak abduction. While no differences were detected in the maximum value of adduction the JIA-P had a lower maximum abduction (JIA-P = 4.2°; CG = 6.0°; *P* <  .05). The absolute ROM from peak adduction to peak abduction was decreased in JIA-P (*P*  <  .01) as well. 

#### 3.2.3. Knee

From the initial contact (*K*_1_) to the flexion peak (*K*_2_) there was no significant difference (Table 2). Knee extension at the end of single support phase (*K*_3_) was significantly reduced (*P*  <  .01). The maximum flexion during swing phase (*K*_4_) was smaller in JIA-P (*P*  <  .001) (Figure 4). Sixteen JIA-P had a bilateral and three a unilateral restriction in the knee extension with a deficit of at least 5° to neutral position, measured in the clinical examination.

#### 3.2.4. Ankle/Foot

**Figure 5:**
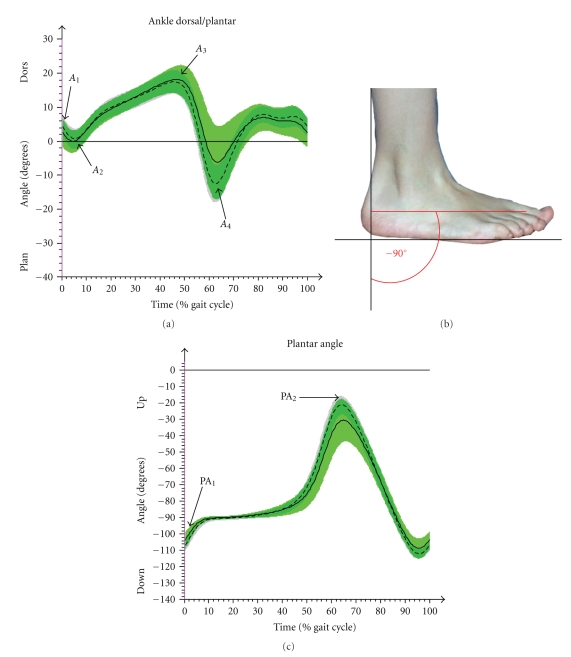
Results of CG (arithmetic mean & SD (- - -)) and JIA-P (arithmetic mean & SD (—)) in time normalized (%) gait cycle in ankle and plantar angle. (a) In ankle (dorsal/plantar flexion), *A*_1_ (initial contact), *A*_2_ (min. value of dorsal flexion in loading response), *A*_3_ (max. dorsal flexion in stance phase), and *A*_4_ (max. plantar flexion while push off or swing) were used for statistical analyses. (b) The plantar angle is the negative angle between the vertical to the ground and the foot longitudinal axis. (c) In plantar angle, PA_1_ (initial contact), PA_2_ (peak in swing phase), and the percentage of time while the foot is flat on the ground (−90° (±2°)) were of interest.

The dorsal flexion of the ankle joint movement throughout the stance phase (*A*_2_-*A*_3_) was increased within JIA-P (+2°) but this was not significant. The following plantar flexion while push off (*A*_3_-*A*_4_) was decreased in JIA-P compared to CG (*P*  <  .01) (Figure 5). A static motion limitation in plantar flexion (less than 50° plantar flexion) was seen bilaterally in 29 patients by the clinical joint assessment.

The plantar angle (Figure 5) was smaller in JIA-P in the initial contact (PA_1_) (*P*  <  .01), as well as in the peak during swing phase (PA_2_) (*P*  <  .001). The roll off behavior was measured by the time while the foot was parallel to the ground (±2°) in stance phase. JIA-P showed a prolonged phase of foot flat. 

### 3.3. Kinetic Results

#### 3.3.1. Ankle Dorsal Flexion Moment and Power

**Figure 6:**
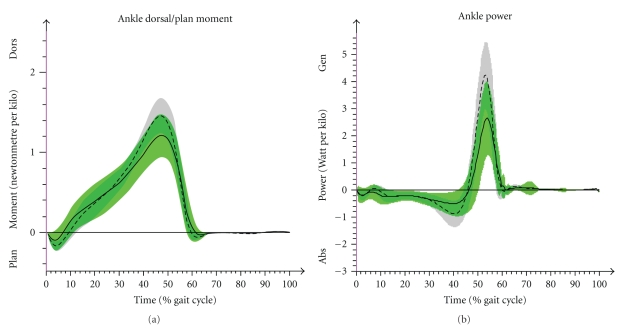
It shows kinetic results of CG (arithmetic mean & SD (- - -)) and JIA-P (arithmetic mean & SD (—)) in time normalized (%) gait cycle of the ankle. (a) Ankle moment (dorsal/plantar flexion). (b) Ankle power.

The maximum peak of kinetic ankle dorsal flexion moment showed smaller values in JIA-P compared to the CG (*P*  <  .001). The same difference was observed for the maximum ankle power during the push off. Values in JIA-P were significantly decreased (*P*  <  .001) (Figure 6).

#### 3.3.2. Vertical and Horizontal Ground Reaction Force

**Figure 7:**
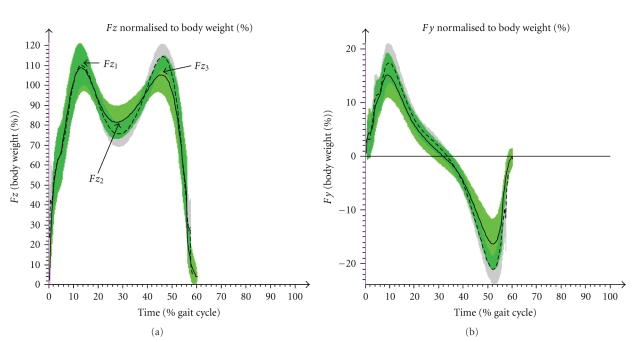
Kinetic results of CG (arithmetic mean & SD (- - -)) and JIA-P (arithmetic mean & SD (—)) in time normalized (%) gait cycle of ground reaction forces (GRF). (a) In the vertical GRF (*F* *z*) the peaks *F* *z*_1_, *F* *z*_3_ and the value *F* *z*_2_ were compared. (b) In the horizontal GRF (*F* *y*) in gait direction the max. peak and the min. peak were used for statistical analyses.

The comparison of the three turning points *F* *z*_1–3_ displayed only in *F* *z*_2_ a statistically significant difference (*P*  <  .05) (Table 3; Figure 7). In the horizontal plane the ground reaction force values of JIA-P were decreased in the push off (*P*  <  .001). Maximum values for the deceleration indicated no differences.

## 4. Discussion

### 4.1. Differences between JIA-P and Normal Gait

Comparison of gait parameters between JIA Patients with a polyarticular pattern of joint involvement of the lower extremities and healthy young individuals showed statistically significant differences. First the decreased walking speed of patients may result from pain, movement restrictions, and compensatory movements but as well from insufficient practice. Decrease of the self-selected walking speed in JIA patients has been observed in other studies as well. There is a statistically significant negative correlation with pain and progressive movement speed in children with JIA [3, 13]. The decreased walking speed is accompanied by a shorter step length of the JIA-P. 

The measured hip extension restriction during single stance phase in JIA-P together with the smaller ROM in the hip (flexion/extension) may be responsible for the shorter step length and slower walking speed [6]. These results fall into place with the clinical examination where one third of the patients had a reduced static hip extension. The increased pelvic tilt may be a compensatory movement of the decreased hip extension as well as the reduced knee extension in single stance phase. Effects of this matter may lead to a higher energy consumption and a reduced leg stability [14].

Götz-Neumann [14] explained the reduced knee extension, that was measured in the JIA-P while single stance phase, as an adaptation to excessive hip flexion during single stance phase or a weak m. gluteus maximus. Furthermore knee pain and hypertonic knee flexors may be responsible for conspicuous reduced knee mobility. Knee joint involvement typically results in muscular imbalance with hypertonia of the hamstring muscles and hypotonia of the m. quadriceps femoris [15, 16]. This leads to a reduced forward progression [6]. The clinical joint assessment revealed knee contractures in 16 patients. But it seems unlikely that this restriction is the reason for the reduced extension during single support phase because the knee extension in initial contact showed normal extension and was equal to the control group.

The decreased maximum knee flexion in swing phase measured in JIA-P may be interpreted as a functionally reduced locomotion and thus be a symptom of knee pain or reduced forward motion of the thigh which is in accordance with the data reported by others [14]. 

The knee flexion during loading response was found to be in normal ranges and is therefore better than expected from the data of a smaller patient group of JIA patients with minor amount of affected joints that we published before [5].

The ankle joint of JIA-P showed an increased (not significant) dorsal flexion during stance phase. This must be interpreted together with the observation of the extended time duration while the foot stands flat on the ground and the strongly decreased plantar flexion during push off. The timing of foot off appears in JIA-P 1.2% of a gait cycle later than in CG. Although the toe off in our investigation groups appeared within the normal range of a gait cycle, the previous facts suggest a more passive and decelerated roll off behavior in patients. These results are supported by the decreased plantar flexion in JIA-P during gait which was confirmed in 29 patients by clinical assessment.

The special character of the sagittal joint movement in the gait of JIA-P with hyperflexion in hip and knee joints and reduced plantar flexion in the ankle may be described as a crouch-like gait. This can be characterized as typical gait for patients with polyarticular JIA. 

The decreased ROM in the contra lateral drop of the pelvis of JIA-P during loading response might be a sign for a compensatory movement. We and others relate this to hip pain [14] and muscle weakness in the m. quadriceps femoris and the gluteal muscles [15]. This motion pattern which was observed in some of the patients corresponds to Duchenne limping. The reduced ROM in pelvic obliquity can be interpreted as stiffness in the pelvis and contributes with a shorter step length and a smaller hip extension (terminal stance phase) to the reduced hip abduction.

Kinetic differences in the lower peaks of ankle joint moments and in ankle power of JIA-P add to a more passive and less dynamic push off compared to controls. This effect also results in the reduced loading of the horizontal ground reaction force (*F* *y*). The vertical force (*F* *z*) divergence between both groups can be explained by the slower walking of patients. 

Although the JIA-P represent a homogeneous sample with similar joint manifestations, standard deviations were increased. This suggests that the disease creates very individual patterns of joint disturbances. 

### 4.2. Therapy Recommendations

The results of the 3d-gait analysis gave new and affirmative arguments that help to recommend sport therapy. Additionally the expertise in pathophysiology and treatment of JIA-P with polyarticular joint pattern was also taken into account for the following suggestions [2, 17, 18]. 

The 3d-gait analysis showed that the patient group suffered from malpositions that can be ascribed to movement restrictions or relieving postures which again can result in further movement restrictions. The main differences compared to controls lay in reduced hip extension, reduced knee extension, and reduced plantar flexion with a passive and decelerated push off of the ankle. Joint restriction goes along with a hypertonic flexor muscle loop and a hypotonic extensor muscle loop. Another study found that the exercise capacity is significantly decreased in a large amount of JIA-P group [19]. Therefore we see that these patients need to practice in different types of physical abilities, mobility, strength, and endurance. It is important that each sector is well balanced. Concerning mobility that means that emphasis must lie on stretching the hypertonic flexor muscle loop but also consider the extensors. To maintain mobility of the pelvic obliquity it seems to be equally important to stretch regularly into hip abduction as well as adduction. Mobility in that region is also important to absorb vertical loadings. Swimming in breaststroke technique could support the functional mobility. 

Pain and inflammation reduce the muscle strength for plantar flexion, knee extension and weaken the gluteal muscles [15]. When inflammation subsides, these muscles should be trained. Therefore the strengthening training for the lower limb should focus on the extensor muscle loop and in a lower dose also for the flexor muscle loop [20]. This could counteract against the measured crouch-like gait.

Continuous sports activity will automatically normalize endurance. So this important part of physical ability can be improved indirectly.

Based on the underlying passivity of the ankle joint and the therefore decelerated progression, as well as on the crouch-like gait, the ability to react on unforeseen situations may be considered to be one of the major limiting factors in JIA-P. In this case, functional joint flexibility is as important as lower limb strength and the ability to coordinate it. 

As JIA tends to run with phases of relapses and remission, we suggest integrating a preventive mobility workout (PMW) for the entire body very close to the present state of an individual patient. A short daily workout including mobility and strengthening exercises could lead to a better functional outcome of the lower limb. This must be analyzed by a longitudinal intervention study.

In general sport activities with a hard and irregular underground like alpine skiing may cause imbalanced movements which result in high strains on the muscular skeletal system. This means a large training stimulus but with higher risk of injury and a possible worse joint prognoses when starting before remission [21]. 

Bicycle riding is a smooth motion with low impact and is regarded as an optimum activity for JIA patients. However, the fixed sitting position may produce movement restrictions especially concerning hip extension. Therefore an individualized stretching program should be included also in a gentle sport like bicycle riding.

If arthritis is located at the lower extremities, optimized training programs should take in exercises with smooth motions and low impact. Motion patterns in joints should develop a large ROM that works towards the extension of knee and hip joints. Examples are the diagonal technique of classic style cross-country skiing, swimming (crawl), or Nordic Walking.

## 5. Conclusion

The results indicate the wide range of disturbances in the mobility of the lower extremities in patients suffering from JIA. 3d-gait analysis has demonstrated to be a powerful tool in quantification of movement abnormalities in patients with JIA. Nearly one third of JIA patients reaching adulthood suffer from limitations in their ability to move [22]. Therefore it is mandatory to enhance the treatment of children with rheumatic diseases especially concerning physio- and sport therapy. Further studies are necessary to provide more detailed data to optimize recommendations for sporting activities.

## Figures and Tables

**Table 1:** Results of spatio-temporal parameters (*= statistically significant).

		Control group (*n* = 20)	mean (SD)	JIA-Patients (*n* = 36)	mean (SD)	*t*-Test
Foot Off	[%]	59.9	1.7	61.1	2.3	.049*
Step Length	[m]	.63	.05	.53	.08	.000*
Dimensionless Step Length		.78	.04	.69	.12	.000*
Walking Speed	[m/s]	1.32	.08	1.06	.17	.000*
Dimensionless Walking Speed		.47	.03	.39	.07	.000*
Step Width	[m]	.09	.02	.12	.04	.010*

**Table 2:** Kinematic parameters in pelvis, knee, and ankle Joint (*: statistically significant; NS: not significant).

		Control group (*n* = 20)	Mean (SD)	JIA-Patients (*n* = 36)	Mean (SD)	*t*-Test
Pelvic Tilt-Average	[°]	10,8	3,9	14,2	5,9	,027*
Pelvic Obliquity (ROM (*P*_1_-*P*_2_))	[°]	3,6	1,2	2,7	1,5	,022*
Pelvic Obliquity ROM	[°]	11,2	5,1	12,4	5,4	NS

Hip Flex/Ext-max. extension	[°]	5,8	5,4	−,7	8,3	,002*
Hip Flex/Ext-max. flexion	[°]	38,2	4,0	39,6	7,4	NS
Hip Flex/Ext-ROM	[°]	44,0	3,3	38,8	5,9	,001*
Hip Abd/Add-max.Abd	[°]	6,0	2,8	4,2	2,0	,009*
Hip Abd/Add-max.Add	[°]	7,8	2,6	7,2	3,0	NS
Hip Abd/Add-ROM	[°]	13,8	3,5	11,4	3,4	,019*

Knee Flex/Ext-*K*_1_	[°]	12,7	3,2	12,4	4,8	NS
Knee Flex/Ext-*K*_2_	[°]	24,6	4,3	23,1	4,9	NS
Knee Flex/Ext-*K*_3_	[°]	8,8	4,4	13,4	4,9	,001*
Knee Flex/Ext-*K*_4_	[°]	65,5	3,0	59,7	6,2	,000*
Knee Flex/Ext (ROM (*K*_1_-*K*_2_))	[°]	12,0	2,3	10,7	3,9	NS
Knee Flex/Ext (ROM (*K*_2_-*K*_3_))	[°]	15,8	3,2	9,7	4,5	,000*
Knee Flex/Ext (ROM (*K*_3_-*K*_4_))	[°]	56,7	3,8	46,4	8,3	,000*

Ankle Dorsi/Plan-*A*_1_	[°]	4,2	2,6	2,6	3,9	NS
Ankle Dorsi/Plan-*A*_2_	[°]	,6	2,8	−,5	3,2	NS
Ankle Dorsi/Plan-*A*_3_	[°]	17,9	2,8	18,9	3,2	NS
Ankle Dorsi/Plan-*A*_4_	[°]	−13,0	4,5	−6,7	10,0	,010*
Ankle Dorsi/Plan-(ROM (*A*_1_-*A*_2_))	[°]	3,6	1,3	3,1	1,8	NS
Ankle Dorsi/Plan-(ROM (*A*_2_-*A*_3_))	[°]	17,4	3,0	19,4	4,0	NS
Ankle Dorsi/Plan-(ROM (*A*_3_-*A*_4_))	[°]	31,0	4,9	25,6	7,9	,008*

Plantar Angle-Initial Contact	[°]	−106,9	2,6	−103,9	4,8	,012*
Plantar Angle-max (swing phase)	[°]	−20,4	4,1	−30,1	11,7	,001*
Foot-Flat (±2°)	[%]	23,7	4,8	27,7	8,0	,045*

**Table 3:** Kinetic parameters of the Ground Reaction Force (GRF) in vertical plane (*z*) and horizontal in gait direction (*y*) as well as the ankle dorsal moment and power (*: statistically significant; NS: not significant).

		Control group (*n* = 16)	Mean (SD)	JIA-Patients (*n* = 35)	Mean (SD)	*t*-Test
GRF(*z*) *P*1	[% BWT]	107,0	15,5	110,6	9,7	NS
GRF(*z*) *P*2	[% BWT]	71,7	9,8	77,4	7,2	,025*
GRF(*z*) *P*3	[% BWT]	111,8	16,4	106,9	6,9	NS

GRF(*y*) *P*1	[% BWT]	17,2	3,7	15,8	3,6	NS
GRF(*y*) *P*2	[% BWT]	−21,3	3,2	−17,2	4,3	,001*

Ankle (max-Dorsi-moment)	[Nm/kg(BWt)]	1,5	,2	1,2	,2	,001*
Ankle (max-Power generation)	[W/kg(BWt)]	4,5	,9	3,0	1,2	,000*
